# Evaluation of the In Vitro Cytotoxic Activity of Caffeic Acid Derivatives and Liposomal Formulation against Pancreatic Cancer Cell Lines

**DOI:** 10.3390/ma13245813

**Published:** 2020-12-19

**Authors:** Magdalena Zaremba-Czogalla, Anna Jaromin, Katarzyna Sidoryk, Agnieszka Zagórska, Marcin Cybulski, Jerzy Gubernator

**Affiliations:** 1 Department of Lipids and Liposomes, Faculty of Biotechnology, University of Wroclaw, Joliot-Curie 14a, 50-383 Wroclaw, Poland; anna.jaromin@uwr.edu.pl; 2 Department of Pharmacy, Cosmetic Chemicals and Biotechnology, Team of Chemistry, Łukasiewicz Research Network-Industrial Chemistry Institute, 8 Rydygiera Str., 01-793 Warsaw, Poland; k.sidoryk@ifarm.eu (K.S.); m.cybulski@ifarm.eu (M.C.); 3 Department of Medicinal Chemistry, Faculty of Pharmacy, Jagiellonian University Medical College, Medyczna 9, 30-688 Krakow, Poland; agnieszka.zagorska@uj.edu.pl

**Keywords:** pancreatic ductal adenocarcinoma, pancreatic cancer, caffeic acid, caffeic acid derivatives, anticancer, cytotoxicity, liposomes

## Abstract

Pancreatic cancer belongs to the most aggressive group of cancers, with very poor prognosis. Therefore, there is an important need to find more potent drugs that could deliver an improved therapeutic approach. In the current study we searched for selective and effective caffeic acid derivatives. For this purpose, we analyzed twelve compounds and evaluated their in vitro cytotoxic activity against two human pancreatic cancer cell lines, along with a control, normal fibroblast cell line, by the classic MTT assay. Six out of twelve tested caffeic acid derivatives showed a desirable effect. To improve the therapeutic efficacy of such active compounds, we developed a formulation where caffeic acid derivative (**7**) was encapsulated into liposomes composed of soybean phosphatidylcholine and DSPE-PEG_2000_. Subsequently, we analyzed the properties of this formulation in terms of basic physical parameters (such as size, zeta potential, stability at 4 °C and morphology), hemolytic and cytotoxic activity and cellular uptake. Overall, the liposomal formulation was found to be stable, non-hemolytic and had activity against pancreatic cancer cells (IC_50_ 19.44 µM and 24.3 µM, towards AsPC1 and BxPC3 cells, respectively) with less toxicity against normal fibroblasts. This could represent a promising alternative to currently available treatment options.

## 1. Introduction

Pancreatic cancer (PC) is one of the most lethal malignancies. In the most recent reports, it ranks seventh in the world and fourth in Europe amongst the leading causes of cancer-related deaths [1,2]. Of PCs 90% constitute pancreatic ductal adenocarcinoma (PDAC), which is one of the most aggressive cancers of the digestive system [3]. A general trend toward an increase of incidence and mortality rates has meant that PDAC is projected to advance as the third leading cause of cancer-related deaths in the USA by 2030 [4]. Despite substantial research efforts and therapeutic improvements, the 5-year survival rate for PC patients remains at 2–9%, while the median survival rate is about 6 months after diagnosis [1]. The reasons behind such bad statistics include the fact that diagnosis is often made at late stages of the disease (due to its long asymptomatic progression) when surgical approaches are not feasible anymore, rapid cancer development and metastasis to other tissues and resistance to conventional approved drugs [5]. PC treatment options are limited and depend on the cancer stage. Due to a late diagnosis, only 15–20% of patients are amenable for surgery. Therefore, conventional systemic cytotoxic treatments, such as chemotherapy and radiotherapy, are the only therapeutic options for the majority of patients. Currently, chemotherapy is mainly based on the use of gemcitabine, albumin-bound paclitaxel (nab-paclitaxel, abraxane) or a multidrug therapy involving a combination of chemotherapeutic agents in the FOLFIRINOX regimen (FOL—folinic acid, 5-FU—5-fluorouracil, RIN—irinotecan and OX—oxaliplatin). However, such therapy does not bring satisfactory results and, moreover, it causes undesirable side effects [6,7,8,9]. In view of this fact, there is a need to find compounds that are safe and efficacious treatments for advanced pancreatic cancer patients. 

Numerous agents isolated from plant materials have been tested on many cancer cell lines. Recent research suggests that caffeic acid (CA; 3,4-dihydroxy cinnamic acid) may be a potentially active anticancer substance [10,11]. CA is a phenolic compound synthesized through secondary metabolism by all plant species, but has been primarily identified in coffee. It is present in many foods, particularly fruits, vegetables, wine, tea and herbs, such as thyme and oregano, and is widely consumed in the human diet [12,13]. It has been confirmed that CA, along with its natural and synthetic derivatives, possesses a variety of pharmacological properties [14], in addition to antibacterial [15,16,17], antiviral [18,19], antimalarial [20,21], anti-inflammatory [22,23], antidiabetic [24], cardioprotective [25] and neuroprotective [26] properties, it has also shown anticancer activity [27,28,29], including activity against pancreatic cancer cells [30,31]. Moreover, it has been reported that CA and its derivates interact synergistically with conventional cytotoxic agents, for example, 5-fluorouracil [32], cisplatin [33], docetaxel and paclitaxel [34]. Various biological studies that have been carried out over the years have revealed that the molecular mechanisms underlying CA anticancer effect are connected with decreasing cell proliferation (antiproliferative effect), increasing intracellular reactive oxygen species (ROS) levels, changing mitochondrial membrane potential, lipid peroxidation and induction of apoptosis [28,35,36]. CA has strong antioxidant activity [13,37] but, under certain conditions, such as the presence of transition metal ions, also shows prooxidant properties [38,39]. CA has the ability to cap endogenous metal ions, for instance, DNA-related copper ions (Cu II). The copper-redox reaction releases reactive oxygen, which causes DNA damage (oxidative base modifications, strand breaks and DNA adducts) and induces lipid peroxidation [27,40,41,42]. Caffeic acid derivatives have also been considered as selective matrix metalloproteinases (MMP-9 and MMP-2) inhibitors [43,44,45], express antiangiogenic activity [46] and suppress DNA methylation [47].

With its broad spectrum of activity and variety of possible pharmacological effects, CA has been widely used as a template for the development of new derivatives with potential therapeutic applications [14]. During previous work, our group synthesized a series of caffeic acid derivatives via a modified Wittig reaction, with variable numbers and positions of free or protected phenolic hydroxyl groups and a modified carboxyl acid terminal group [48].

In this study, as a continuation of previous work, we investigated the possible anticancer activity of twelve resynthesized CA derivatives against two pancreatic cancer cell lines: AsPC-1 and BxPC-3 (cells of metastatic and primary origins, respectively), and NHDF (normal human dermal fibroblasts) cells. Using the MMT assay, we observed specific antiproliferative effects for some of the derivatives tested and we were able to select two of the most active ones for further study. While the poor water solubility of these compounds is a challenge for their clinical application, we encapsulated them into liposomes for delivery to tumor cells (to improve their bioavailability and increased stability). In general, drug delivery systems have been reported to enhance the efficacy and reduce the toxicity of anticancer drugs. Long circulating nanocarriers, such as PEGylated liposomes, are well-studied and very promising carriers, which facilitate preferential drug accumulation in tumors via the enhanced permeability and retention (EPR) effect, allowing the lowering of the required dose of the drug and reducing the level of toxic side-effects. The other advantages of using liposomes are the protection of the drug from the surrounding environment and the prevention of its early degradation, resulting in a longer circulation time in the blood. All these features result in an improvement in the therapeutic index of the encapsulated drugs [49,50,51,52]. The PEGylated liposomes generated during this study were characterized for their physicochemical properties (including size, shape, zeta potential and stability at 4 °C), the ability to promote hemolysis of human erythrocytes and their cytotoxicity and cellular uptake by tumor cells. We wished to investigate whether the compounds encapsulated as liposomal formulations had potent anticancer activity against the pancreatic cancer cell lines AsPC-1 and BxPC-3 and were less toxic to normal cells.

## 2. Materials and Methods 

### 2.1. Chemicals and Reagents

RPMI-1640, alpha-MEM media and the normal human dermal fibroblast cell line (NHDF) were purchased from Lonza (Lonza, Warsaw, Poland), while fetal bovine serum (FBS) was from Biomedia (EuroClone by Biomedica, Piaseczno, Poland), GlutaMAX™ (L-alanyl-L-glutamine dipeptide in 0.85% NaCl) and 100× antibiotic-antimycotic were purchased from Life Technologies (Gibco/Life Technologies, Warsaw, Poland). The BxPC-3 cell line was purchased from the American Tissue Culture Collection (ATCC, Manassas, VA, USA). The AsPC-1 cell line was kindly provided by the Institute of Immunology and Experimental Therapy (Wroclaw, Poland). Nile Red (9-diethylamino-5H-benzo[alpha]phenoxazine-5-one), DAPI (2-(4-amidinophenyl)-6-indolecarbamidine dihydrochloride) and MTT (3-(4,5-dimethylthiazol-2-yl)-2,5-diphenyltetrazolium bromide) were from Sigma-Aldrich (Poznan, Poland), chloroform, methanol and DMSO from Archem (Kamieniec Wroclawski, Poland). Soya phosphatidylcholine (SPC) was purchased from Lipoid GmbH (Ludwigshafen, Germany). 1,2-distearoyl-sn-glycero-3-phosphoethanolamine-N-[amino(polyethylene glycol)-2000] (DSPE-PEG_2000_) was obtained from Northern Lipids (Vancouver, BC, Canada).

### 2.2. Caffeic Acid Derivatives Synthesis, pK_a_ and Log D Calculation

Caffeic acid derivatives were resynthesized using a method reported previously [48]. The negative logarithm of the acid dissociation constant (pK_a_) and the decimal logarithm distribution coefficient (Log D) were calculated using MarvinSketch software (version20.12.0, ChemAxon Ltd., Cambridge, MA, USA).

### 2.3. Cell Culture

In vitro cell culture procedures were performed under aseptic conditions and cultures were maintained in an Innova CO-180 incubator (New Brunswick Scientific, Edison, NJ, USA). Pancreatic cancer cells, namely, BxPC-3 (primary pancreatic tumor) and AsPC-1 (from ascites of a patient with pancreatic cancer) cells, were cultured in RPMI-1640 medium supplemented with 10% heat inactivated fetal bovine serum (FBS), GlutaMAX™ and antibiotic-antimycotic. NHDF cells were cultured in alpha-MEM medium supplemented with 10% heat-inactivated FBS, GlutaMAX™ and antibiotic-antimycotic. Culture was carried out at 37 °C in a humidified atmosphere containing 5% CO_2_. 

### 2.4. Liposome Preparation

Caffeic acid derivatives encapsulated liposomes were prepared using the dry lipid film and extrusion method. A 1 mL solution of 10 mg of total lipids in chloroform (SPC:DSPE-PEG_2000_ 95:5 mol/mol) were mixed with 1 mg of caffeic acid esters in methanol. Subsequently, solvents were removed from the samples via evaporation under a stream of nitrogen-gas to form a homogeneous lipid film on the flask wall and dried for 2 h at low pressure using a Savant Modulyo lyophilizer (Thermo Fisher Scientific, Waltham, MA, USA). Then, the lipid film was suspended by addition of 1 mL of sterile normal saline (0.9% NaCl), with mixing, until it was completely hydrated. The liposomal suspensions were extruded 6 times through nucleopore polycarbonate filters (Whatman, Maidstone, UK) with pore sizes of 100 nm, using a Thermobarrel Extruder (10 mL Lipex extruder, Northern Lipids, Burnaby, BC, Canada). During the extrusion procedure, nonencapsulated compounds were separated from the loaded liposomes and were deposited on the surface of the polycarbonate filter. Empty liposomes without CA derivatives were prepared in the same manner. In the case of Nile Red loaded liposomes, 50 µL of Nile Red solution (1 mg/mL) was added additionally to the total lipids.

### 2.5. Determination of Compound Encapsulation Efficiency (EE)

The lipid concentration was determined by the Stewart assay [53]. The amount of (**7**) was determined spectrophotometrically at 290 nm and was calculated from the appropriate calibration curve according to the method described previously [54]:

EE (%) = CAf(mg/mL)/Lf(mg/mL)/CAi(mg/mL)/Li(mg/mL) × 100(1)

where CAi(mg/mL)/Li(mg/mL) represents the (caffeic acid derivate)-to-lipid ratio at the point when the compound was mixed with the liposomes, and CAf(mg/mL)/Lf(mg/mL) is the final (caffeic acid derivate)-to-lipid ratio of the liposomes.

### 2.6. Determination of Particle Size and Zeta Potential

The average particle sizes and zeta potentials of the loaded and control (empty) liposomes were determined by dynamic light scattering at room temperature (25 °C) using a ZetasizerNano-ZS particle size analyzer (Malvern Instruments Ltd., Malvern, UK). Before measurements, all formulations were diluted 100-fold with 0.9% NaCl. Measurements were performed in triplicate. The polydispersity index (PdI) was obtained using the instrument’s built-in software. 

### 2.7. Analysis of the Liposomal Morphology by Transmission Electron Microscopy (TEM)

Transmission electron microscopy (TEM) was employed to observe the structure of liposome samples. Initially, liposomes were diluted and introduced onto a copper grid and dried at room temperature. Subsequently, samples were stained with 2% uranyl acetate and again dried at room temperature. The analysis of liposomal morphology was performed using a TESLA BS 540 transmission electron microscope (Brno, Czech Republic).

### 2.8. Preliminary Stability Study

The size, PdI and zeta potential of 7-L and control liposomes were measured immediately after preparation (t = 0) and after storage at 4 °C for 35 days.

### 2.9. MTT Assay

The effect of CA and its derivatives on cell viability was determined using a quantitative colorimetric MTT assay adapted from Mosmann [55]. Cells were seeded in 96-well plates (5 × 10^3^/well), in appropriate complete cell culture medium, for 24 h. The cells were treated with caffeic acid derivatives (in the range of 18.75–150 μM), DMSO (an equivalent volume of DMSO was used as a negative control, maximal concentration was 0.4% v/v) or liposomal formulations for 72 h. The medium containing tested chemicals was removed and MTT solution (working solution: stock 0.5 mg/mL was 10 times diluted in medium) was added to the wells, and the plates were incubated for a further 3 h. Subsequently, the MTT solution was replaced with DMSO (50 μL/well) to dissolve the purple formazan crystals. Absorbance was measured at 560 nm with a reference wavelength of 670 nm on an Asys UVM 340 Microplate Reader (Cambridge, UK). Results were expressed as the percentage of surviving cells, with respect to the control (the untreated cells) calculated as:

Cell Viability (%) = (AT/AC) × 100(2)

where:

AT = Absorbance of the treatment well (treated cells);
AC = Absorbance of the control well (not treated cells).

IC_50_ values were calculated using GraphPad Prism for Windows (GraphPad Software, La Jolla, CA, USA). The degree of selectivity of the CA derivatives against pancreatic cancer cells was expressed by the selectivity index (SI) where:

SI = IC_50_ normal cells/IC_50_ cancer cells (3)

### 2.10. Red Blood Cell Hemolysis Assay

Hemolytic activity was estimated by measurement of hemoglobin release from human erythrocyte suspensions after incubation with liposomes, as previously described [56]. 7-L was added in a volume corresponding to 200 µM concentration in a sample (L was added in an identical volume). The study protocol was approved by the Bioethics Commission at the Lower Silesian Medical Chamber (1/PNHAB/2018).

### 2.11. Cellular Uptake 

Cellular uptake of Nile Red loaded liposomes by BxPC3 cells was assessed by fluorescence microscopy. Cancer cells were seeded onto glass cover slides, placed in 24-well culture plates. After 48 h incubation, cell culture medium was replaced with medium containing Nile Red labeled liposomes. The cells were incubated at 37 °C for 2 h. Subsequently, cells were washed three times with PBS (37 °C), to remove excess liposomes and fixed for 20 min in 4% paraformaldehyde, washed with PBS and stained with DAPI. Slides were analyzed using a Leica TCS SP8 confocal microscope (Leica-Microsystems, Mannheim, Germany) with a HC PL APO CS2 63× oil objective. To excite Nile Red and DAPI, 561 nm and 405 nm lasers (Leica-Microsystems, Mannheim, Germany) were used, respectively.

### 2.12. Statistical Analysis 

Data were presented as mean ± standard deviation. Statistical analyses were made using GraphPad Prism software with a one-way ANOVA (Prism 7 for Windows) and Dunnett’s multiple comparisons test. A *p*-value equal or less than ≤0.05 was considered as statistically significant. 

## 3. Results

### 3.1. Characteristics of Compounds *(**1**)*–*(**12**)*

The modification strategy of CA was related to reduction in the number (compounds (**1**)–(**3**), (**7**)–(**9**)), position (compounds (**4**) and (**10**)) or addition (compounds (**5**), (**6**) and (**11**)) of free phenolic hydroxyl groups. Moreover, the terminal carboxylic group was modified by introduction of an ester bond (methyl ester, compounds (**1**)–(**6**)) or reduction of the carbonyl function (acetyl group, compounds (**7**)–(**11**)). The structure modification introduced to compound (**12**) relied on the substitution of the 3-hydroxyphenyl by the 3-methoxy group and a change of the terminal carboxylic group to methyl ketone (Table 1).

The acid-base property of a molecule, pK_a_, is the key parameter determining its solubility, absorption, distribution, metabolism and elimination (ADME-tox). Thus, pK_a_ has become of great importance, because the transport of compounds into cells and across other membranes is a function of their physicochemical properties, and of the pK_a_ of the molecules. Moreover, based on the pK_a_ value of the molecule, it is possible to estimate the pH, which ensures the maximum solubility of the molecule in water, determined by the ionized form of the drug, or its maximum solubility in a non-polar environment, in the non-ionized form. Compounds (**1**)–(**3**), (**7**)–(**9**) and (**12**) had a single pK_a_ value in the range of 8.87–9.51, while compounds (**4**) and (**10**) each exhibited two pK_a_ states, namely, pK_a1_ = 9.40, pK_a2_ = 10.40 and pK_a1_ = 8.69, pK_a2_ = 10.04, respectively, whereas compounds (**5**) and (**11**) had three pK_a_ states (Table 1). Based on the pK_a_ values of these series of compounds, it can be concluded that they all underwent slight ionization in the physiological pH range, which reduces their solubility in a polar environment. The overall ratio of the ionized and unionized molecular forms between the polar/non-polar phases at pH 7.4 was described as the decimal logarithm of distribution coefficient (Log D). The estimated Log D values of compounds (**1**)–(**12**) were in the range of 2.07–2.84, which indicated their moderate lipophilicity. It is noteworthy that Log D values of compounds (**1**)–(**12**) only differ slightly from each other, despite the huge differences in their structures (e.g., a lack of one hydroxyl group in compounds (**1**)–(**3**), or the introduction of a ketone group in compounds (**7**)–(**9**) versus the CA core structure). The key difference in the 1–6 and 7–12 groups of compounds is that the first contains the methyl ester of caffeic acid derivatives while, in the second group, the ester moiety prone to hydrolysis is replaced with a stable ketone carbonyl group bonded to two carbon atoms.

### 3.2. In Vitro Biological Activity against Pancreatic Cell Lines

**Figure 1:**
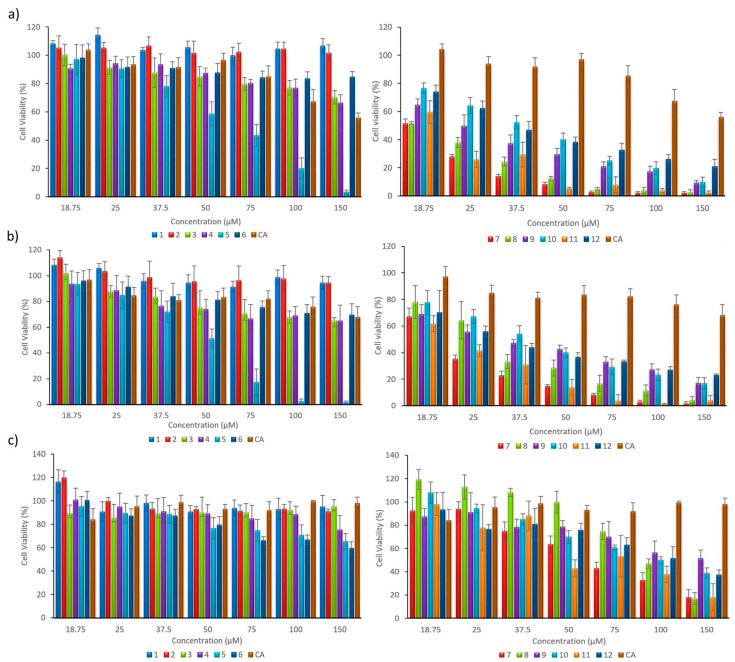
Cytotoxic effects of caffeic acid (CA) and its derivatives (compounds **1**–**6** and **7**–**12**), on two human pancreatic cancer cells lines AsPC1 (**a**), BxPC3 (**b**) and control NHDF (**c**) cells, determined by the MTT assay, after 72 h of incubation.

The purpose of this study was to obtain a preliminary insight into the anticancer activity of a series of caffeic acid derivatives. In order to determine the in vitro cytotoxicity of the resynthesized compounds, the activity of CA and twelve derivatives (Table 1), were screened against two human PC cell lines, BxPC3 and AsPC1, derived from primary and metastatic tumors, respectively. Since we were also interested in evaluating the degree of selectivity of the synthetized derivatives, we decided to use normal human dermal fibroblasts as control, healthy, cells. This made it possible to determine the specificity of the tested substances, in terms of their selectivity index towards pancreatic cancer cells. The cytotoxicity of the examined compounds was estimated using the classic MTT assay, which is based on the detection of the activity of oxidoreductive enzymes (mainly mitochondrial succinate dehydrogenase), which are present only in living, metabolically active cells [55]. During the experiments, cells were incubated for 72 h in the presence of increasing concentrations of CA and the series of CA derivatives, in the range of 18.75–150 μM. Tested compounds were dissolved in DMSO, a solvent broadly used for drug tests, which, in the concentrations tested (≤0.4%), did not influence cell viability (data not shown). Untreated cells were tested as the control group. The results obtained for each cell line are presented in Figure 1.

Six out of twelve tested compounds appeared to be more active than CA. In the case of the first group of tested compounds, the in vitro cytotoxicity test showed that, compared to caffeic acid and other tested compounds, compound (**5**) had the highest cytotoxic activity against pancreatic cancer cells, with low toxicity to control fibroblasts (Figure 1, left). The results presented in Figure 1 indicate that all compounds included in the second group (**7**–**12**) exhibit dose-dependent, anticancer activity against both pancreatic cancer cell lines in the concentration range tested. What is more, the cytotoxic activity of the derivatives was greater, compared to CA. Additionally, their cytotoxicity against the normal cell line was lower compared to cancer cells, which is reflected also in their selectivity indices (Table 2).

Quantitatively, the biological activity of the test compounds was expressed as the IC_50_ (inhibitory concentration), defined as the concentration that inhibits the growth of cells by 50%, relative to the control (untreated cells), which is assumed to be 100%. Results regarding the cytotoxicity and selectivity obtained for the biological active compounds are summarized in Table 2.

Based on the obtained IC_50_ values, the most active derivatives were indicated. From the first group, the most active compound was (**5**), with IC_50_ values of 42.47 µM and 46.58 µM, respectively, for the AsPC1 and BxPC3 pancreatic tumor cells, and an IC_50_ of 98.82 µM for the NHDF normal line (Table 2). The SI demonstrates the selectivity index and, the greater the SI value is, the more selectively this compound acts and, theoretically, would be safer as a drug. An SI value higher than 2 is considered as selective to malignant cells over normal, healthy, cells [57]. For compound (**5**), SI values were higher than 2, proving its selective action towards both pancreatic cell lines.

For the second group, IC_50_ concentrations for the tested malignant cells were in the range of 18–37 µM. The data showed that compounds (**7**) and (**11**) had the lowest IC_50_ values, compared with the other compounds (18.70 µM, 22.38 µM and 18.35 µM, 21.72 µM towards AsPC1 and BxPC3 cells, respectively). Moreover, both showed specificity of action towards cancer cells, which was confirmed by their respective SI indexes (in the range of 2.73–3.36; Table 2). 

### 3.3. Preparation and Physical Characterization of CA Derivate Loaded Liposomes

Low aqueous solubility of CA derivatives makes administering them at therapeutic doses very difficult. To overcome this problem, we decided to encapsulate the most active representatives, compounds (**7**) and (**11**) into liposomes. The selected CA derivates were encapsulated within the phospholipid bilayer of liposomes composed of soybean phosphatidylcholine (SPC) and 1,2-distearoyl-sn-glycero-3-phosphoethanolamine-N-[amino(polyethylene glycol)-2000] (DSPE-PEG_2000_) (95:5 mol/mol). The prepared formulations were subsequently characterized. Encapsulation efficiency was found to be 93% for the compound (**7**) formulation. Due to the high levels of compound precipitation encountered with compound (**11**) during liposome preparation, it was decided to terminate this part of the study and, therefore, we continued our work with compound (**7**) alone. We investigated the physicochemical characteristics of this liposomal formulation in terms of size, PdI, zeta potential, shape and preliminary stability at 4 °C. As shown in Table 3, dynamic light scattering (DLS) results showed that the diameter of liposomes was around 137.33–147.4 nm. 

**Figure 2:**
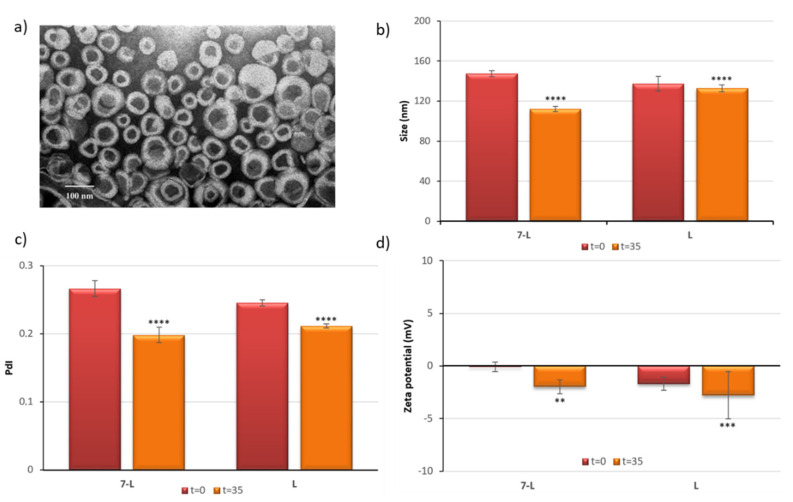
Transmission electron microscopy image of 7-L liposomes (**a**), changes of size (**b**), polydispersity index PdI; (**c**) and zeta potential (**d**) of the (**7**) loaded liposomes (7-L) and control liposomes (L) just after preparation (t = 0 days) and stored at 4 °C for 35 days. A star (*) show the statistical significance, where ∗∗ *p* < 0.01, ∗∗∗ *p* < 0.001, and ∗∗∗∗ *p* < 0.0001 are significantly different from time 0.

The macroscopic appearance of the liposomal suspension was milky and yellow. To visualize the shapes and morphology of liposomes, transmission electron microscopy was used. TEM image showed that the 7-loaded liposomes (7-L) were mostly spherical and had a regular shape (Figure 2). Additionally, we also conducted preliminary stability studies at 4 °C. It is worth noting that, after 5 weeks of storage, no dramatic changes, such as visible aggregation or precipitation, in the appearance of the tested liposomes occurred. Storage stability was also examined by measurements of size, PdI and zeta potential, as shown in Figure 2, under the same conditions. Stored liposomes showed some changes in all parameters, more visible in the case of loaded vesicles. Overall, the obtained results confirmed the satisfactory stability of the liposomal suspensions during storage.

### 3.4. Hemolysis

Hemolysis assays were performed to test the membrane disruptive activity of the 7-L formulation at a concentration of (**7**) corresponding to 200 µM. No activity was demonstrated, and the data for both loaded and unloaded liposomes were at the level of the results obtained for the mechanical control of the test (erythrocytes in PBS buffer). This suggests that the developed formulations are non-toxic to red blood cells at the concentration tested.

### 3.5. Assessment of Liposome Toxicity on the Cell Viability of Human Pancreatic Cancer Cell Lines

**Figure 3:**
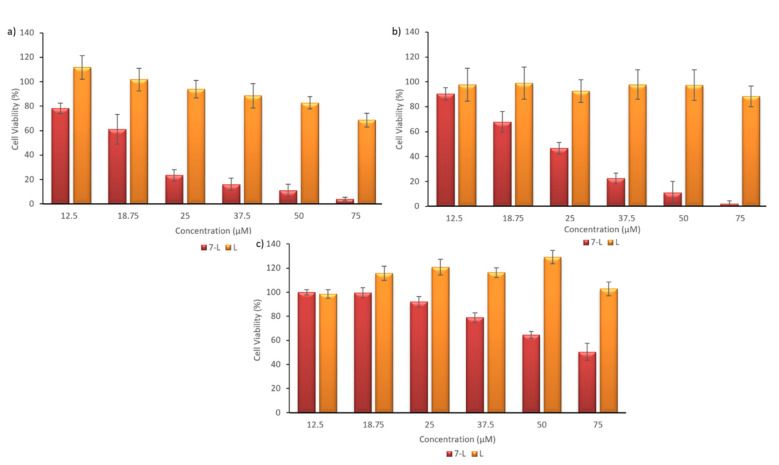
Pancreatic cell viability assessed by the MTT assay. Concentration–cell viability curves for (**a**) AsPC1 (**b**) BxPC3 and NHDF (**c**) cells treated with different amounts of (**7**) loaded liposomes (7-L) and control vesicles without drug (L) for 72 h.

To evaluate the anticancer potential of the 7-L liposomal formulation, we investigated its in vitro cytotoxicity against two human pancreatic cell lines (AsPC1 and BxPC3). During experiments, cells were incubated for 72 h with the 7-L liposomal formulation and empty control liposomes (without drug) at the same amounts as the drug loaded ones. The data indicated that the viability of cells treated with 7-L, was similar to that observed with the free compound (19.44 µM and 24.3 µM, towards AsPC1 and BxPC3 cells, respectively). Moreover, 7-L also showed specificity against cancer cells (SI indexes: 3.32 and 2.65, in the case of AsPC1 and BxPC3 cells, respectively; Table 4). We observed that control liposomes also had a toxic effect at the highest vesicle concentrations. This concerns especially the AsPC1 cell line (Figure 3). 

### 3.6. Cellular Uptake

**Figure 4:**
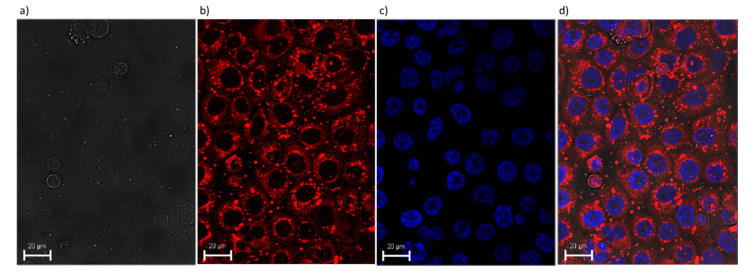
Visualization of the intracellular uptake of Nile Red loaded liposomes by BxPC3 pancreatic cancer cells. Transmitted light (**a**), Nile Red fluorescence signal (red) (**b**), DAPI fluorescence (blue) (**c**) and the merged (**d**) image of cells observed after 2 h incubation with labeled liposomes. Scale bar = 20 μm.

The next step was to evaluate cellular uptake of the liposomes. For this purpose, liposomes loaded with fluorescent Nile Red dye were used. Nanoparticle labeling with this hydrophobic dye, which fluoresces preferentially in non-polar environments, is commonly used for bioimaging studies [58,59,60,61]. Cellular uptake and localization were investigated by fluorescence microscopy. For chromosome staining, a nucleic-acid specific fluorophore, DAPI, was used. The Nile Red fluorescence signal surrounded the DAPI nuclear stain. Images of the cells observed under transmitted light and fluorescence channels after 2 h incubation with labeled liposomes revealed that labeled vesicles were effectively internalized within BxPC3 cells (Figure 4).

## 4. Discussion

To date, pancreatic cancer is a serious global problem, being one of the most aggressive and lethal malignancies because of difficulties in diagnosis, early metastasis, recurrence and resistance to chemotherapy [2]. Therefore, there is an urgent need to improve PC patient survival rates, which can be achieved through the development of better diagnostic tools and the discovery of new, effective and safe drugs. Such drugs may be natural plant substances or their synthetic analogues, created by structural modification [62,63]. In our previous work we described the synthesis method for a series of novel CA-derived compounds [48]. The present study aimed to investigate the efficiency of these compounds for their potential as anticancer agents towards pancreatic cancer cell lines. So far, many studies demonstrated that CA and its derivatives (among numerous other potential therapeutic properties) are compounds with potent anticancer effects. In vitro experiments have revealed their cytotoxic properties inter alia on breast [64,65], ovarian [66,67], colon [68,69,70,71], hepatoma [72,73,74], melanoma [75,76,77], prostate [78] and pancreatic [30,31,79] cancer cell lines.

In this study we tested the antiproliferative effect of twelve CA derivates against two pancreatic cancer cell lines, namely, AsPC1 and BxPC3. Six of the compounds were found to be more active than non-modified CA. From the first tested group, compound (**5**) was interesting, in the context of higher activity compared to the others. This compound has three OH groups. Previous research suggests that OH groups undergo deprotonation and can react with Cu ions and, consequently, generate ROS and form a covalent adduct with DNA [27]. It is known that pancreatic cancer cells lines are very sensitive to compounds generating free radicals, due to the high basal level of ROS in these cells [80,81,82]. This is especially relevant to metastatic cells, where this phenomenon is amplified, due to their high metabolic rate (the Warburg effect) and a higher level of transition metal ions stored in the cytoplasm of these cells (involved in free radical reactions). This may, in part, account for the specificity of these derivatives for pancreatic cancer cells.

Among all of the tested compounds, (**7**) and (**11**) exhibited the highest effect on cell viability towards AsPC1 and BxPC3 cells. Moreover, both compounds also showed specificity of action towards cancer cells, which was confirmed by their SI indexes. This desired cytotoxic effect, in addition to weaker activity towards normal healthy fibroblasts, which are widely used in the literature as a control cell line [83,84], suggests that CA derivatives could constitute very promising targets for PC treatment. What is worth emphasizing, the most active compound, namely 4-(2’, 3’, 4’-trihydroxyphenyl)-3 (E)-buten-2-one, numbered as compound (**11**), is a new compound described by us in patent application. In the literature, similar results were observed for another CA derivative, caffeic acid phenethyl ester (CAPE). CAPE is a well-known natural CA derivative, the main active component of propolis. It has confirmed cytotoxic, antiproliferative and anticancer properties, and specifically inhibits NF-κB (nuclear factor kappa-light-chain-enhancer of activated B cells) and 5-lipoxygenase activity [28,85]. Studies have revealed that CAPE, at a concentration of 25 μg/mL (87 μM), reduced the proliferation rates of the MIAPACa-2 and PANC-1 pancreatic cancer cell lines by 65.7% and 93.0%, respectively, after a 48 h incubation period [79]. Similar inhibition was observed for BxPC3 cells (80.4%) treated with 10 μg/mL (35 μM) CAPE [31]. Moreover, researchers have also proven that the combined use of CAPE and cabazitaxel, a compound used for treating PC patients resistant to paclitaxel, enhanced the cabazitaxel effect in vivo and in vitro [70]. Interestingly, recently published data suggest that CAPE can protect normal pancreatic tissue against damage associated with the administration of cisplatin [86].

The intensity of a drug’s biological activity primarily depends on its bioavailability. Low water solubility of the CA derivates, similar to many synthetic and herbal compounds, is a limiting feature of their applicability as pharmaceutical products. A possible strategy to overcome this problem is the development of suitable delivery systems, which may provide better bioavailability and facilitate delivery of the compound to the target site at the appropriate concentration in vivo. In the literature, a number of different delivery strategies and carriers for CA and derivates have been described [87], e.g., solid lipid nanoparticles [88], calcium phosphate nanocomposites [89] and cyclodextrin-based hydrogels [17]. We chose liposomes as the optimal carrier system for our studies, since we were able to solubilize hydrophobic molecules that partition within the lipid bilayer. Several liposomal formulations carrying caffeic acid and their derivatives have also been described in the literature. For example, caffeic acid was successfully encapsulated in liposomes for topical applications, to improve skin penetration through the stratum corneum and to enhance transdermal delivery, thereby developing a promising drug delivery vehicle to slow down photooxidative damage to the skin [90].

We encapsulated compounds (**7**) and (**11**) as the most active representatives of the series of molecules tested, into liposomes composed of soybean phosphatidylcholine (SPC) and DSPE-PEG_2000_ in a ratio of 95:5 (mol/mol). Pegylation has been proven to prolong the blood circulation time of liposomes and to reduce mononuclear phagocyte system uptake [91], while SPC is a natural biocompatible element. Compound (**7**) is hydrophobic, with a high affinity to the liposomal bilayer, in contrast to compound (**11**), which we failed to encapsulate. In the future, we will focus on elaboration of liposomes with more rigid bilayers. The most important part is to improve on the release ratio of the encapsulated compound so it can be made bioavailable to cancer cells, while maintaining the favorable pharmacokinetics of the compound at the same time. Pancreatic cancer is characterized by the high amount of extracellular matrix present in the cancerous lesion, hindering drug penetration into the tumor mass [92]. Carriers, characterized by their nano size and stealth effect (long circulation time) are able to accumulate to some degree in the PDA tissue by the so-called EPR effect. This effect can be further increased by several approaches, such as, hyperthermia, pretreatment with TNFα, PEGylated hyaluronidase, a low dose of angiogenesis inhibitors or losartan [52,93,94,95,96,97]. 

The presence of (**7**) within the liposome bilayer caused significant changes, resulting in a larger size and PdI of 7-L, compared to empty liposomes. The encapsulation efficiency of compound (**7**) was high (93%). Moreover, as expected, the 7-L liposomes in the TEM images appeared slightly smaller than the sizes determined from DLS measurements. This is due to the fact that DLS measurements were performed for liposomes in solution, while the TEM technique was used to estimate the size of dried vesicles, which were smaller than in the hydrated state.

Another aspect that was analyzed in our study was the stability of the obtained vesicles. Stability is a very important factor that must be carefully considered during the design and development of new formulations. A possible explanation for the trends observed during the stability studies is the influence of encapsulated (**7**) upon the liposome bilayer. In order to determine if both loaded and unloaded vesicles disrupt human red blood cells, hemolysis experiments were conducted. These experiments were performed at a concentration of (**7**) that represented a concentration almost ten times greater than the estimated IC_50_ for both cancer cell lines. As expected, no hemolytic activity was observed in our studies, supporting the conclusion that both nanoformulations are safe and compatible with red blood cells.

Further, to investigate internalization and the cytotoxic effect of the 7-L liposomes, we performed a cellular uptake study and monitored cytotoxicity via the MTT assay. We confirmed that, after 2 h of incubation, Nile Red labeled liposomes were effectively internalized by pancreatic cancer cells. We observed that empty liposomes also had a toxic effect at the highest vesicle concentrations. Such an effect for plain SPC has been already described in the literature [98] and can be explained by an increase in the fluidity of the mitochondrial membrane and the release of cytochrome c and reduction of the cholesterol-to-phospholipid ratio and increase of lipid disorder in the cell membrane, which can lead to reduction in cell viability [98,99]. 7-loaded liposomes inhibited cell proliferation in a dose-dependent manner. Under our experimental conditions, the IC_50_ for liposomal (**7**) was comparable to free compound (**7**) administered in DMSO. However, the use of liposomes as carriers enables application of molecules characterized by poor water solubility, such as (**7**), to biological fluids, like blood, which, consequently, should provide higher bioavailability of such compounds and result in an increase in the desired biological effects after intravenous administration. 

In summary, this study revealed the potential of a CA derivative loaded in a liposomal formulation as a suitable candidate that could become a promising anticancer drug formulation for the treatment of human pancreatic cancer. 

## Figures and Tables

**Table 1:** List of tested compounds, their chemical structures, pK_a_ and Log D values.

Compound	Structure	pK_a_	Log D pH 7.4
(**1**)	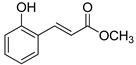	9.22	2.65
(**2**)	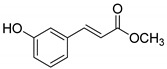	9.40	2.84
(**3**)	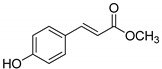	9.40	2.84
(**4**)	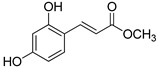	10.40 9.40	2.55
(**5**)	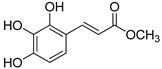	14.49 10.96 8.82	2.26
(**6**)	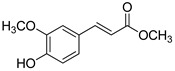	9.87	2.59
(**7**)	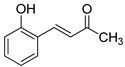	8.87	2.65
(**8**)	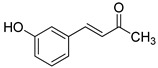	9.41	2.66
(**9**)	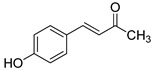	9.04	2.65
(**10**)	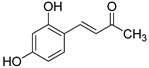	10.04 8.69	2.36
(**11**)	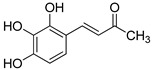	14.48 10.43 8.66	2.07
(**12**)	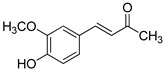	9.51	2.41
CA	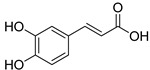	12.69 9.28 3.45	−1.67

**Table 2:** IC_50_ values calculated from the MTT assay for biologically active caffeic acid derivatives. Selectivity index (SI) = IC_50_ of NHDF/IC_50_ of cancer cell lines.

Compound	AsPC1 IC_50_ (µM)	BxPC3 IC_50_ (µM)	NHDF IC_50_ (µM)	AsPC1 SI	BxPC3 SI
(**5**)	42.47 ± 3.57	46.58 ± 3.47	98.82 ± 9.56	2.33	2.12
(**7**)	18.70 ± 0.24	22.38 ± 2.52	61.15 ± 1.95	3.27	2.73
(**8**)	19.22 ± 0.72	30.47 ± 4.55	79.70 ± 6.30	4.14	2.61
(**9**)	25.62 ± 2.17	28.06 ± 3.36	59.18 ± 11.57	2.31	2.11
(**10**)	35.30 ± 1.28	34.11 ± 1.74	54.57 ± 2.25	1.54	1.60
(**11**)	18.35 ± 0.74	21.72 ± 1.51	61.73 ± 10.5	3.36	2.84
(**12**)	37.96 ± 6.12	29.94 ± 5.13	51.48 ± 1.20	1.36	1.72

**Table 3:** Liposome characterization.

Liposomes	Diameter (nm)	PdI	Zeta Potential (mV)
7-L	147.40 ± 2.90	0.27 ± 0.02	−0.08 ± 0.44
L	137.33 ± 7.19	0.24 ± 0.01	−1.69 ± 0.60

7-L—(**7**) loaded liposomes; L—control, empty liposomes.

**Table 4:** IC_50_ values calculated from the MTT assay for the liposomal formulations. Selectivity Index (SI) = IC_50_ of NHDF/IC_50_ of cancer cell line.

Liposomes	AsPC1 IC_50_ (µM)	BxPC3 IC_50_ (µM)	NHDF IC_50_ (µM)	AsPC1 SI	BxPC3 SI
7-L	19.44 ± 0.83	24.30 ± 1.29	64.50 ± 2.83	3.32	2.65

7-L—(**7**) loaded liposomes.
